# SH2D5 promotes lung adenocarcinoma cell metastasis and triggers EMT via activating AKT signaling pathway

**DOI:** 10.1371/journal.pone.0316432

**Published:** 2024-12-30

**Authors:** Licheng Du, Wenjia Ren, Linjun Liu, Haojia Zhu, Ke Xu, Yubai Zhou

**Affiliations:** 1 Department of Biology, College of Chemistry & Life Science, Beijing University of Technology, Chaoyang, Beijing, China; 2 NHC Key Laboratory of Biosafety, National Institute for Viral Disease Control and Prevention, China CDC, Changping, Beijing, China; Sichuan University, CHINA

## Abstract

Lung adenocarcinoma (LUAD) is the most common histological subtype of lung cancer, characterized by a high incidence in late stages, high mortality rate, and poor prognosis. Src Homology 2 Domain Containing Protein 5 (SH2D5) is a mammalian-specific, uncharacterized scaffolding protein, and its role in LUAD remains unclear. In the present study, we investigated the function and potential mechanisms of SH2D5 in the progression of LUAD. We found aberrant expression of SH2D5 in LUAD tissues and cells, and its high expression is closely associated with poor prognosis in LUAD patients. Through loss-of-function and gain-of-function experiments, we revealed that overexpression of SH2D5 promotes the proliferation and migration abilities of lung adenocarcinoma cells. Gene set enrichment analysis (GSEA) revealed that SH2D5 positively regulates the epithelial-mesenchymal transition (EMT) process in lung adenocarcinoma cells. Additionally, we found that regulating the expression of SH2D5 influenced the phosphorylation levels of AKT, and the rescue experiments with AKT pathway activators/inhibitors partially reversed the tumor progression and EMT processes induced by SH2D5. In summary, our study demonstrated that SH2D5 promotes the migration and EMT process of LUAD cells through the AKT signaling pathway, suggesting that SH2D5 may serve as a crucial potential target for the treatment of metastatic LUAD.

## Introduction

Lung cancer is the most common cancer worldwide and the leading cause of cancer-related deaths worldwide, seriously affecting human health, according to the latest estimates of global cancer burden data published by the World Health Organization’s International Agency for Research on Cancer (IARC) [1]. Lung adenocarcinoma (LUAD) as the most common type of lung cancer, which accounts for about 40% of all lung cancer cases [2]. Moreover, LUAD has the highest incidence of metastasis, which can spread rapidly to lymph nodes, contralateral lung, and multiple distant organs [3]. Despite tremendous advances in multimodal treatment strategies including targeted therapy, immunotherapy, radiotherapy, and surgical resection in recent decades, the five-year overall survival rate for LUAD is currently less than 20%, with distant metastasis resulting in a survival rate of approximately 7% [4–6]. Patients with metastatic LUAD are mostly unresponsive to currently available treatments and have a high frequency of recurrence after treatment. Thus, it is particularly urgent and crucial to identify novel molecular targets to control the invasion and distant metastasis of LUAD.

Epithelial-mesenchymal transition (EMT) is a cellular plasticity procedure of lineage transition between epithelium and mesenchyme [7,8]. The polarized epithelial cells can lose their adhesive properties and acquire mesenchymal cell phenotypes by the process of EMT, which is characterized by the enhanced mesenchymal markers (snail and slug) and abated epithelial markers (E-cadherin) [9–11]. Accumulating evidence suggests a relationship between the EMT process and the invasion and metastatic progression of malignant tumor cells [12,13]. In most cancers, cancer cells develop EMT, and the cells lose their cell polarity and cell-cell adhesion, gaining the ability to migrate and invade [10,14,15], allowing them to eventually achieve the purpose of proliferation and metastasis after extravasation [16]. Recently, it was documented that EMT can be regulated through different molecular mechanisms and pathways [10]. AKT is a critical component in many processes and has been shown to regulate the EMT process [17]. Previous studies have shown that this pathway promotes the invasion and migration properties of malignant cells by inducing EMT, either directly or in combination with other pathways [18,19].

Src homology 2 domain containing protein 5 (SH2D5) is a mammalian endemic, uncharacterized adaptor protein that localizes to human chromosome 1 and is highly enriched in the brain at the transcriptional level and may be involved in regulating synaptic plasticity by controlling Rac-GTP levels [20]. Structurally, SH2D5 contains an N-terminal PTB domain and a C-terminal SH2 domain, whose PTB domain binds to the breakpoint cluster region (BCR) protein and mediates Rac1-GTP levels involved in synaptic plasticity [20,21]. The expression level of SH2D5 in the liver tissue of HBV-associated hepatocellular carcinoma (HCC) patients was significantly higher than that in adjacent tissues. In addition, HBV infection can cause an increase in SH2D5 levels, and Hepatitis B virus X protein (HBx) plays an important role in inducing SH2D5 expression, and HBx can stimulate SH2D5 expression through NF-κ B and c-Jun kinase pathways [22]. Similarly, SH2D5 has also been found to be aberrantly expressed in LUAD, and its high expression may be inversely correlated with immune invasion [23]. However, the mechanism of SH2D5 in LUAD still needs to be elucidated.

In the present study, we aimed to investigate the biological function of SH2D5 in the progression of LUAD and its potential molecular mechanisms. First, through bioinformatics analysis and cellular experiments, we found that SH2D5 is upregulated in LUAD, and its abnormal expression is associated with the survival rate of LUAD patients. Subsequently, by conducting loss-of-function and gain-of-function experiments, we revealed that the overexpression of SH2D5 promotes the proliferation and migration of lung adenocarcinoma cells. Additionally, gene set enrichment analysis (GSEA) and rescue experiments further demonstrated that SH2D5 enhances the EMT process via the AKT signaling pathway, thereby regulating the metastatic capability of LUAD cells. This study may reveal the new points into the metastasis of LUAD and indicate SH2D5 may be a potential therapeutic target for LUAD metastasis.

## Methods and materials

### Bioinformatic analysis

The expression data for the lung adenocarcinoma (LUAD) cohort were obtained from The Cancer Genome Atlas (TCGA) program via the Genomic Data Commons Data Portal (https://portal.gdc.cancer.gov/). Corresponding clinical information was sourced from TCGA and a peer-reviewed publication [24]. The expression levels of SH2D5, quantified in Transcripts Per Kilobase Million (TPM), were extracted and subsequently transformed to log2(TPM + 1) for normalization. Then, differential expression analyses, including both unpaired and paired comparisons, were conducted to evaluate the expression differences of SH2D5 between the LUAD samples and normal samples, as well as between LUAD tumors and their corresponding adjacent normal tissues. These analyses were performed using the Wilcoxon rank sum test within the R statistical computing environment (version 4.2.1). A p-value threshold of < 0.05 was adopted to denote statistical significance. For survival analysis, LUAD patients were stratified into high- and low-expression subgroups based on the median expression level of SH2D5. Overall survival (OS) and disease-specific survival (DSS) were assessed using the Cox proportional hazards regression model, as implemented in the survival R package (version 3.3.1). To elucidate the underlying mechanisms of SH2D5 in LUAD progression, Gene Set Enrichment Analysis (GSEA) was performed using the hallmark gene sets (h.all.v2022.1.Hs.symbols.gmt) from the Molecular Signatures Database (MSigDB) with the clusterProfiler R package (version 4.4.4). Adjusted p-values (p.adj) or false discovery rates (FDR) < 0.05 were set as the criterion for statistical significance.

### Cell lines and cell culture

The human lung adenocarcinoma cell lines H1299 (p53 null), HCC827 (EGFR 19 deletion) and A549 (KRAS mutant) were obtained from the National Collection of Authenticated Cell Cultures (Shanghai, China). The normal human bronchial epithelial cell lines 16HBE were provided by the Chinese Academy of Cell Resource Center (Shanghai, China). H1299, HCC827 were cultured in Roswell Park Memorial Institute 1640 (RPMI-1640) medium (Thermo Fisher Scientific, USA) containing 10% fetal bovine serum (FBS; Thermo Fisher Scientific, USA) and 1% penicillin/streptomycin (P/S; Thermo Fisher Scientific, USA). A549, 16HBE were cultured in Dulbecco’s modified Eagle’s medium (DMEM; Thermo Fisher Scientific, USA) supplemented with 10% FBS and 1% P/S. All cells were incubated in a humidified incubator at 37°C and 5% CO_2_.

### Lentivirus and plasmid transfection

Three small hairpin RNAs (shRNAs) targeting SH2D5 (shSH2D5-1, shSH2D5-2 and shSH2D5-3, with the sequences 5’-GCCTTCAGGGTCTCAAGATCT-3’, 5’-GCATACTCTACTCCACCTGGT-3’ and 5’-AGCTCTCTCAGAACGTCCATG-3’) and their negative control (shNC, with the sequence 5’-GGTTCTCCGAACGTGTCACGT-3’) lentiviral vector were obtained from Beijing tsingke Biotechnology Company (Beijing, China). The lentiviral transfection process was conducted according to the Protocol provided by Tsingke. Then, cells were selected with 2.5 μg/ml puromycin for 2 weeks. To establish SH2D5 overexpressing cell lines, the pcDNA3.1 plasmid was procured from Hunan Fenghui Biotechnology Company (Hunan, China). Then the full length of SH2D5 was sub-cloned into pcDNA3.1. Cell transfection was performed using the jetPRIME® transfection reagent (Polyplus, France) according to the manufacturer’s instructions, and the efficacy of transfection was assessed by western blotting. The AKT inhibitor, MK-2206 (Sellcek, USA) and the AKT activator, SC79 (Beyotime, Shanghai, China), were added for 24h to the cells at a concentration of 10 μM.

### Cell proliferation and colony formation assay

Cell growth rate was assessed using the Cell Counting Kit-8 method (CCK-8, Beyotime, China) following the manufacturer’s protocol. Cells (2 × 10^3^ cells per well) were seeded in 96-well plate and incubated for 24, 48, or 72h. After each time point, each well was supplemented with 10 μL CCK-8 reagent to culture for additional 2h. The absorbance was detected by a microplate auto-reader (Perkin Elmer, USA) at 450 nm.

For the colony formation assay, 700 cells were seeded per well in 6-well plates in culture medium containing 10% FBS for about 2 weeks. The cell colonies were fixed with 4% paraformaldehyde for 20 min and then stained with crystal violet for 15 min. After staining, each well was washed with water and air-dried. Capture images of the stained colonies using a camera, and use ImageJ to count the number of colonies in each well.

### Wound healing migration assay

For wound-healing assay, the cells were seeded in 6-well plates and cultured until 90% confluence. The cells were scratched with a 10 μL sterile pipette tip in the middle of the well, washed gently with Phosphate Buffered Saline (PBS, Thermo Fisher Scientific, USA) to remove the scratched cells, and cultured in FBS-deficient medium. The wound widths were measured microscopically at different time points and photographed at 0h and 24h/48h, respectively. Calculate and compare the wound healing rates between different sample groups. The assay was repeated three times.

### Transwell migration and invasion assays

Transwell chamber (8 μm, Corning Costar, USA) with or without Matrigel matrix (Corning, USA) was used to assess the invasion or migration ability of LUAD cells. For the migration assay, cells were seeded on the upper side of transwell chamber (5 × 10^4^ cells per chamber) fill with 200 μl basal medium, while the lower well was filled with 600 μl medium containing 10% FBS. In the invasion experiment, Matrigel matrix was diluted 1:9 with precooled basal medium at 4°C and coated on the bottom of the transwell chamber. Then, the transwells were incubated in the incubator for more than 4 hours to allow the matrix to denature and solidify with heat. The next procedure was the same as the migration experiment (1 × 10^5^ cells per chamber). After incubating for 48h, the cells were fixed with 4% paraformaldehyde for 15 min and then stained with crystal violet for 15 min. Photographs of three random regions were taken, and the number of migrated cells was counted with a microscope (Olympus, Tokyo, Japan).

### Western blotting

Total protein was extracted cells using cell lysis buffer for Western and IP (Beyotime, Shanghai, China) containing protease and phosphatase inhibitor cocktail (Beyotime, Shanghai, China). Bicin-choninic Acid (BCA) protein quantification kit (Shanghai Yazyme Biotechnology, Shanghai China) was used to detect the protein concentration. Equal amounts of protein were separated by 10% sodium dodecyl sulfate–polyacrylamide gel electrophoresis (SDS-PAGE) for 1.5 h and transferred onto polyvinylidene fluoride (PVDF) membranes (Millipore, USA). After blocking with QuickBlock^™^ Blocking Buffer (Beyotime, Shanghai, China) for 30 min, the membranes were washed with Tris-buffered saline with Tween-20 (TBST) solution three times for 10 min each time and incubated overnight at 4°C with the indicated primary antibodies. And then the membranes were washed by TBST three times, further incubated with horseradish peroxidase (HRP)-conjugated secondary antibody goat antirabbit IgG H&L (Beyotime, Shanghai, China) at room temperature for 1h. The visualization of bands was performed with hypersensitive enhanced chemiluminescence (ECL) kit (Beyotime, Shanghai, China) in Tanon 5200 chemiluminescence imaging system (Tanon, Shanghai, China). Analyze the captured images using ImageJ. Use the software to quantify the intensity of the bands corresponding to both the target protein and the endogenous control protein (β-tubulin). The intensity of the target protein bands will be normalized to that of β-tubulin to calculate relative expression levels, ensuring accurate quantification and comparisons across samples.

Primary antibodies were purchased from Cell Signaling Technology (E-Cadherin, 1:1000, #3195; Snail, 1:1000, #3879; Slug, 1:1000, #9585), Affinity Biosciences (SH2D5, 1:1000, #DF4498; Tubulin beta, 1:2000, #AF7011), Abways Technology (Akt(pan) 1/2/3, 1:1000, #CY5561; Phospho-Akt (Ser473), 1:1000, #CY6569).

### Statistical analysis

All data were shown as mean ± standard deviation from three independent experiments. Difference comparisons between two groups were performed by using Student’s t-test. One-way Analysis of Variance (ANOVA) method was used for comparisons between multiple groups. The following p-values indicate statistical significance: *p ≤ 0.05, **p ≤ 0.01, ***p ≤ 0.001, and ****p ≤ 0.0001. All statistical analyses were performed using GraphPad Prism version 9.0.0. for Windows, GraphPad Software, San Diego, California USA, www.graphpad.com.

## Results

### SH2D5 is aberrantly expressed in LUAD and is associated with poor prognosis

**Fig 1:**
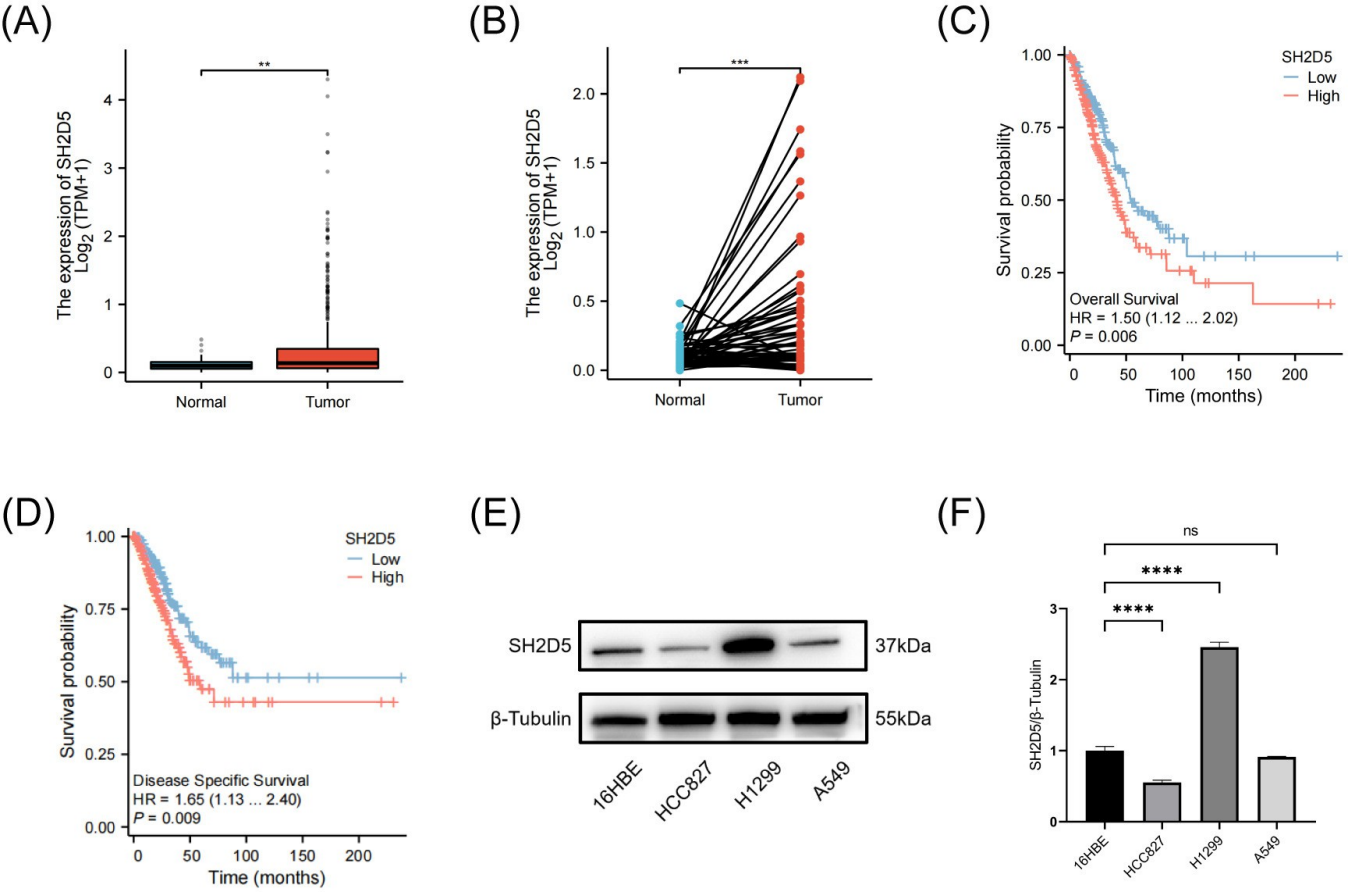
High SH2D5 expression in lung adenocarcinoma (LUAD) is associated with poor prognosis. (A) The expression level of SH2D5 in 539 SH2D5 samples and 59 normal samples in The Cancer Genome Atlas (TCGA) database. (B) The expression of SH2D5 in 58 paired TCGA tumors and corresponding adjacent noncancerous tissues. (C, D) Overall survival and disease specific survival in low and high SH2D5 expression patients in TGCA cohort. (E, F) Western blot analysis of SH2D5 expression levels in 16HBE and LUAD cell lines. **p ≤ 0.01, ***p ≤ 0.001, ****p ≤ 0.0001 and ns p > 0.05.

We analyzed the expression of SH2D5 in LUAD using the TCGA data sets. Results indicated that SH2D5 was significantly upregulated in LUAD samples compared with normal lung tissues (Fig 1A). Meanwhile, SH2D5 was also highly expressed in paired LUAD samples compared with corresponding normal lung tissues (Fig 1B). We next explored the prognostic implications of SH2D5 expression in the TCGA-LUAD database. The results are consistent with previous research [23], showing that patients with higher expression of SH2D5 in LUAD have poorer overall survival (OS) and disease-specific survival (DSS) compared to patients with lower expression of SH2D5 (Fig 1C and 1D). Finally, to gain a more comprehensive understanding of the expression patterns of SH2D5 in lung adenocarcinoma cells, we selected three cell lines that represent distinct genetic backgrounds. As shown in Fig 1E and 1F, the expression of SH2D5 was higher in H1299 and downregulated in HCC827, compared to the normal human bronchial epithelial 16HBE cells. Taken together, these observations confirmed that SH2D5 expression is aberrantly expressed during LUAD progression and increased expression of SH2D5 was associated with the malignant process of LUAD.

### Upregulation of SH2D5 promotes LUAD cell proliferation, migration and invasion

**Fig 2:**
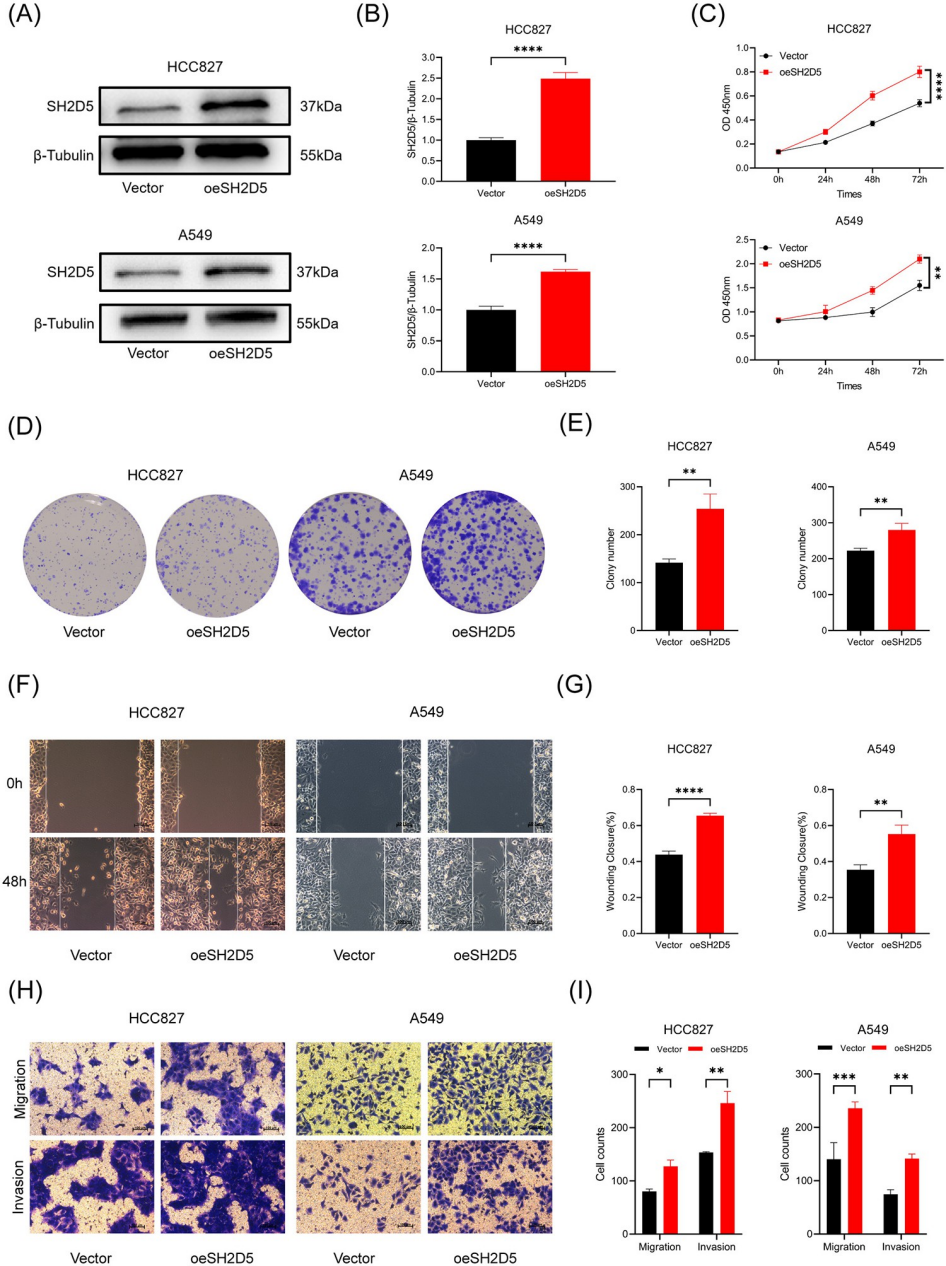
Overexpression of SH2D5 promotes lung adenocarcinoma (LUAD) cell proliferation, migration and invasion. (A, B) Transfection efficiencies after overexpression of SH2D5 in HCC827 and A549 cells determined by western blot. (C-E) The proliferation ability of vector cells and oeSH2D5 cells in HCC827 and A549 was detected by CCK8 and colony formation assays. (F-I) The wound healing and transwell assays indicated the migration and invasion abilities of HCC827 and A549 with vector cells and oeSH2D5 cells. Scale bar = 100 μm; *p ≤ 0.05, **p ≤ 0.01, ***p ≤ 0.001, and ****p ≤ 0.0001.

To evaluate the biological functions of SH2D5, we performed gain-of function studies in two LUAD cell lines, A549 and HCC827. The overexpression efficiencies were confirmed by western blot analysis (Fig 2A and 2B). As shown in Fig 2C–2E, CCK8 and colony formation assays indicated that overexpressed SH2D5 significantly promotes the proliferation of A549 and HCC827 cells compared to that in the control group. Similarly, the wound healing and transwell assays suggested that SH2D5 overexpression significantly promotes cell migration and invasion abilities compared to the control group (Fig 2F–2I). Hence, these outcomes indicate that the upregulation of SH2D5 promotes cell proliferation, migration and invasion.

### Knockdown of SH2D5 inhibits LUAD cell migration, invasion, and proliferation

**Fig 3:**
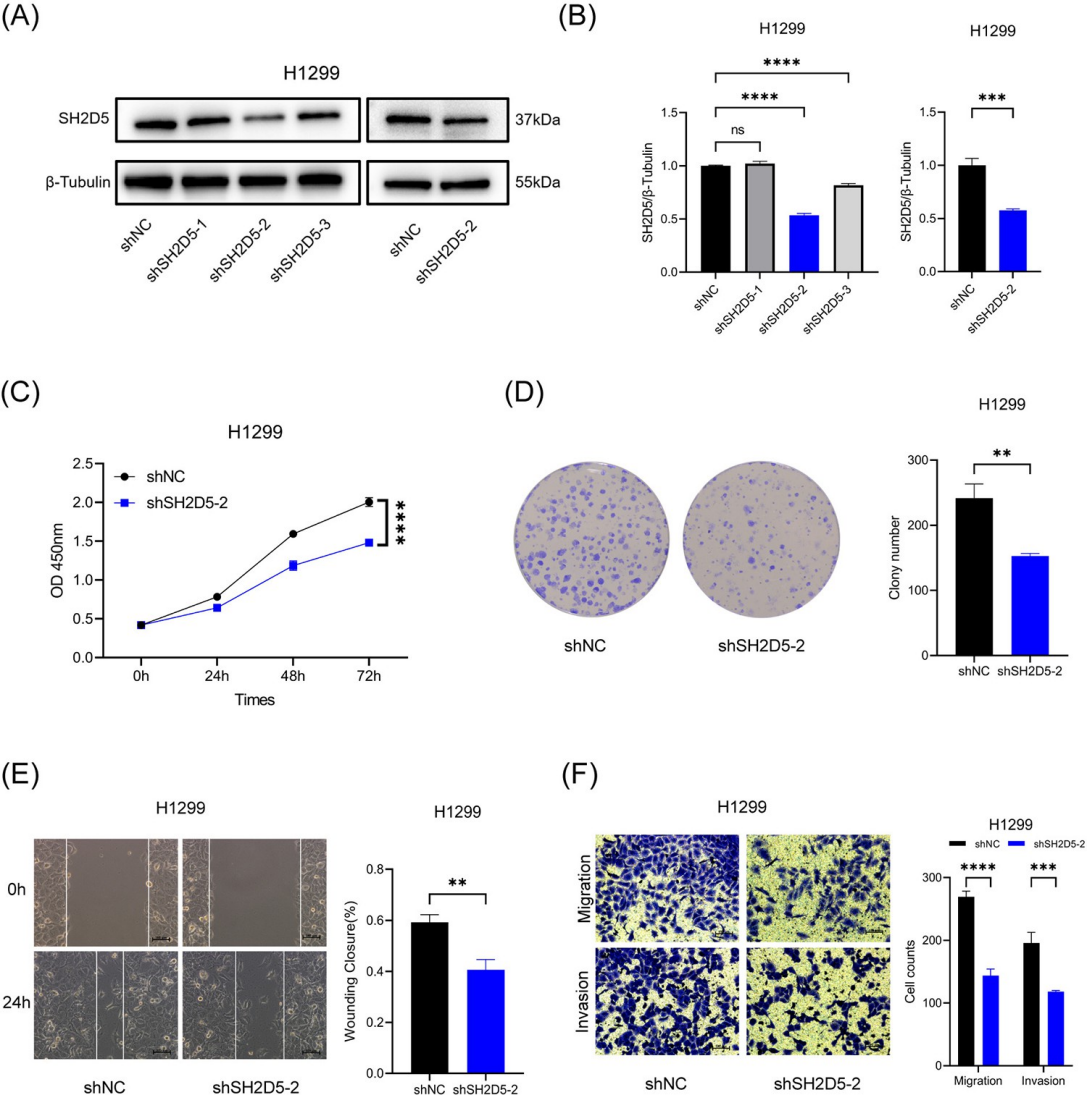
SH2D5 knockdown inhibits lung adenocarcinoma (LUAD) cell proliferation, migration and invasion. (A, B) The western blot analysis was employed to detect SH2D5 knockdown efficiency of transfection in H1299 cells. (C, D) The CCK8 and colony formation assays were used to reveal the cell proliferation in SH2D5- knockdown H1299 cells. (E F) Wound healing and transwell assays were used to detect the cell migration or invasion in SH2D5- knockdown H1299 cells. Scale bar = 100 μm; **p ≤ 0.01, ***p ≤ 0.001, ****p ≤ 0.0001 and ns p > 0.05.

To further explore the role of SH2D5 in LUAD, the expression of SH2D5 was knocked down in LUAD cell line, H1299. The knockdown efficiency was confirmed by western blot assays (Fig 3A and 3B). The results of CCK8 and colony formation assays revealed that the reduction of endogenous SH2D5 decreased the proliferative ability of H1299 (Fig 3C and 3D). Meanwhile, transwell assays and wound healing assays were performed to explore the function of SH2D5 in cell migration and invasion. The results showed that downregulation of SH2D5 reduced cell migration and invasion compared to the control cells (Fig 3E and 3F). Together, these results demonstrated that knockdown of SH2D5 effectively inhibits the proliferation, migration and invasion of LUAD cell. So, SH2D5 could serve as a potential molecular target in LUAD development.

### SH2D5 activates EMT in LUAD cells

**Fig 4:**
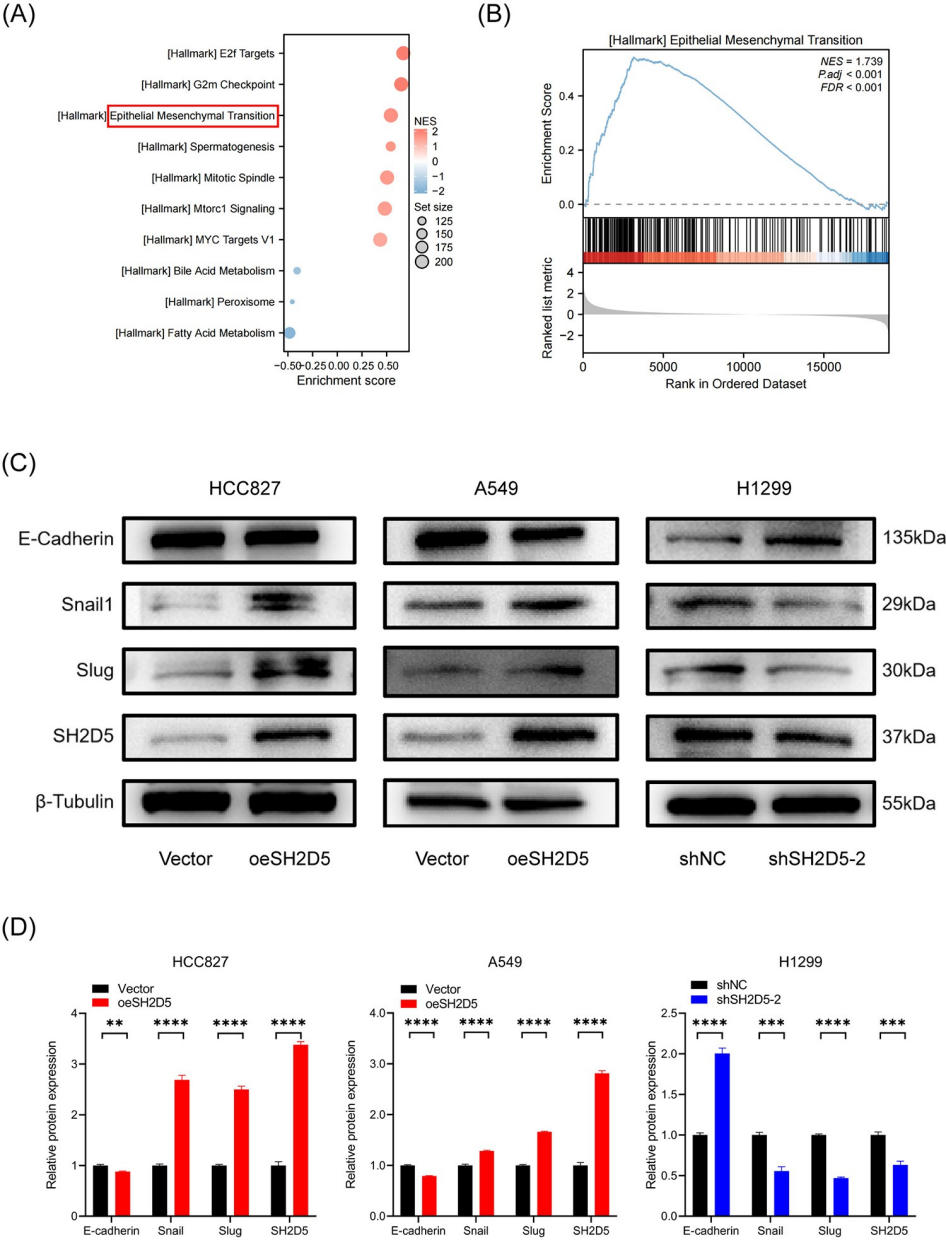
SH2D5 regulates epithelial-mesenchymal transition (EMT) in lung adenocarcinoma (LUAD) cells. (A, B) Gene Set Enrichment Analysis (GSEA) was used to identified the potential regulatory pathways between high and low SH2D5 expression groups. NES, normalized enrichment score. (C, D) The expression of EMT‐related genes in LUAD cells with SH2D5 overexpression or knockdown determined by Western blot analysis. **p ≤ 0.01, ***p ≤ 0.001, and ****p ≤ 0.0001.

EMT plays a critical role in tumorigenesis and metastasis [25]. Through public databases, we analyzed the potential regulatory pathways of SH2D5 in LUAD. Based on the median SH2D5 gene expression, LUAD patients were divided into SH2D5 high expression and low SH2D5 expression groups, GSEA was used to reveal the dysregulated cancer characteristics in each group. The result showed that the HALLMARK EMT pathway was significantly activated in the high expression group (Fig 4A and 4B).

To directly investigate whether SH2D5 could activate EMT in LUAD, we detected the expression of EMT‐related markers, including E‐cadherin, Snail and Slug in protein levels, western blot results revealed that overexpression of SH2D5 in both A549 and HCC827 cells significantly increased the expression of Snail and Slug. Notably, while the overexpression of SH2D5 inhibited E-cadherin protein expression in A549 cells, this inhibition was not apparent in HCC827 cells. Conversely, knockdown of SH2D5 in H1299 cell correspondingly increased the expression of E-cadherin and repressed the expression of Snail and Slug at protein levels (Fig 4C and 4D). These data clearly demonstrate that SH2D5 promotes the development of EMT in LUAD cells.

### SH2D5 promotes EMT of LUAD cells via AKT signaling pathway

**Fig 5:**
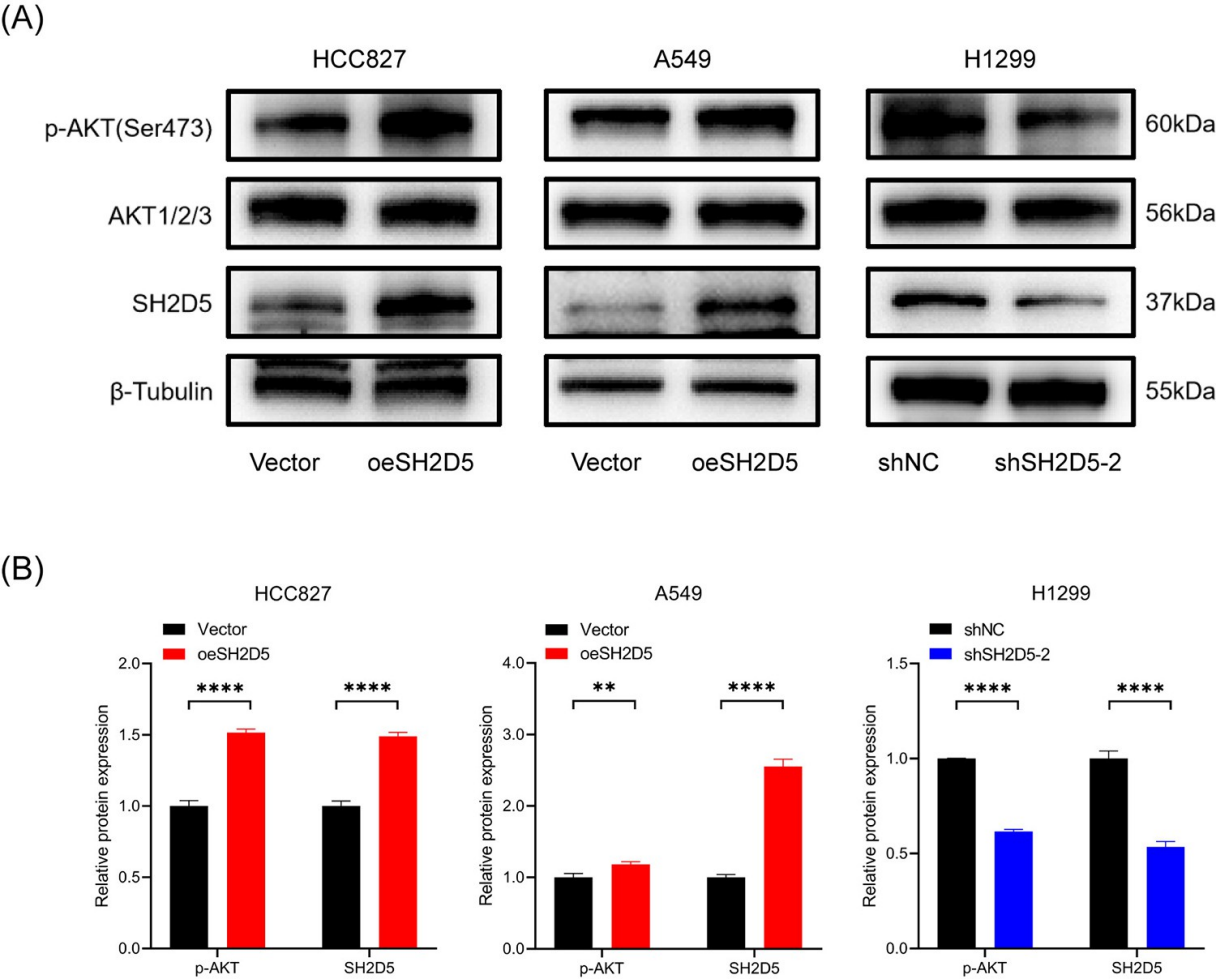
SH2D5 induces AKT pathway in lung adenocarcinoma (LUAD) cells. (A, B) The expression of AKT pathway genes in LUAD cells with SH2D5 overexpression or knockdown determined by Western blot analysis. **p ≤ 0.01, and ****p ≤ 0.0001.

Thereafter, we continued to delve deeper into the potential molecular mechanisms by which SH2D5 regulates the metastasis of LUAD. The AKT signaling pathway plays a crucial role in both the development and progression of lung cancer [26]. A variety of cellular processes such as survival, proliferation, migration and metastasis, are regulated by the AKT pathway in lung cancer [27]. Any change in the components of this pathway may result in lung cancer progression and facilitate tumor metastasis. Therefore, we hypothesized that SH2D5 might regulate the metastasis of LUAD through the AKT signaling pathway. We explored the influence of SH2D5 on the activation of the phosphorylation-AKT signaling pathway in LUAD cells. Western blot analysis found that overexpression of SH2D5 in HCC827 and A549 led to activation of phosphorylation‐AKT in Ser473; correspondingly, SH2D5 knockdown in H1299 cells reduced the phosphorylation‐AKT (Fig 5A and 5B). In summary, these data suggest that SH2D5 activates the AKT signaling pathway in LUAD.

**Fig 6:**
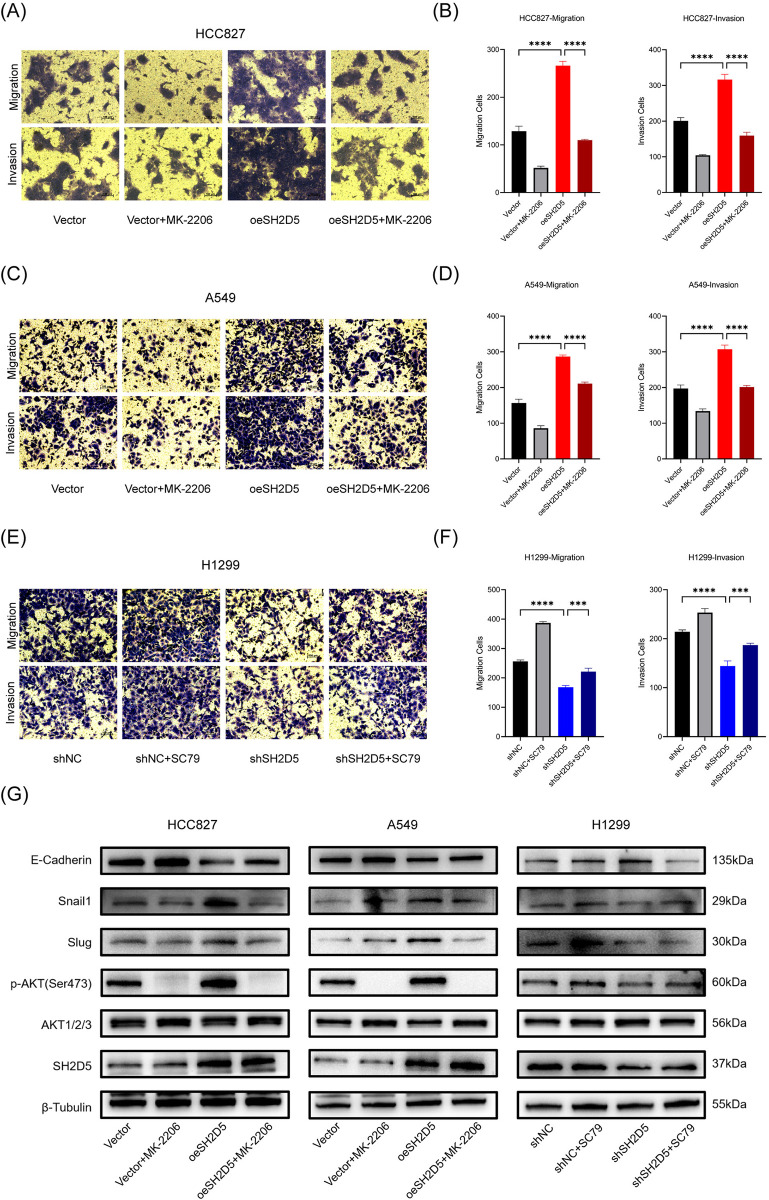
SH2D5 promotes epithelial-mesenchymal transition (EMT) by regulating AKT pathway in lung adenocarcinoma (LUAD) cells. (A-F) Transwell migration and invasion assays performed in overexpression / knockdown of SH2D5 in LUAD cells treated with or without MK-2206 / SC79 (10 μM) treatment. (G) Western blot analysis was used to detect the relative expression levels of phosphorylated AKT, AKT, E-cadherin, Snail and Slug in SH2D5-overexpression cells treated with MK-2206 (10 μM) and SH2D5-knockdown cells treated with SC79 (10 μM) with respective control cells. Scale bar = 100 μm; ***p ≤ 0.001, and ****p ≤ 0.0001.

Furthermore, we further investigated whether the AKT signaling pathway is involved in the EMT process to regulate the metastasis of LUAD. In our study, we utilized the AKT-specific inhibitor (MK-2206, 10μM) and activator (SC79, 10μM) to suppress or activate the AKT signaling pathway in lung adenocarcinoma cells with either overexpression or knockdown of SH2D5, in order to investigate whether the effects of SH2D5 could be reversed. As expected, the inactivation of the AKT pathway partially inhibited the enhanced migratory and invasive abilities induced by the overexpression of SH2D5 in HCC827 and A549 cells (Fig 6A–6D). Conversely, the activation of the AKT pathway partially rescued the attenuated migratory and invasive capabilities caused by the knockdown of SH2D5 in H1299 cells (Fig 6E and 6F). Furthermore, western blot analysis revealed that the treatment with MK-2206 or SC79 partially reversed the changes in EMT-related proteins induced by the overexpression or knockdown of SH2D5 in LUAD cells (Fig 6G). These findings led to the speculation that the regulation of EMT in LUAD cells could be done by SH2D5 through the activation of the AKT pathway.

## Discussion

Lung cancer is one of the leading causes of cancer-related deaths globally, and lung adenocarcinoma (LUAD) is the most common histological subtype of lung cancer. In recent years, substantial progress has been made in the treatment of LUAD patients, including the development of targeted therapies and immunotherapies [28]. However, since some LUAD patients are diagnosed at a stage when metastasis has already occurred, rendering them ineligible for targeted treatments, the 5-year survival rate for LUAD patients remains relatively low [5]. Therefore, the identification of molecules that can serve as both prognostic biomarkers and therapeutic targets for the regulation of LUAD metastasis has become increasingly urgent and critical.

Src Homology 2 Domain Containing Protein 5 (SH2D5) is a mammalian endemic, uncharacterized scaffold protein. Previous studies have found that in HBV-related hepatocellular carcinoma, the expression of SH2D5 is abnormally upregulated, and it promotes the proliferation and metastatic ability of cells [22]. This suggests that SH2D5 may play a crucial role in the metastasis of carcinomas. However, the role of SH2D5 in LUAD is unknown. In this study, by analyzing the TCGA-LUAD dataset using online bioinformatics tools, we demonstrated that the expression of SH2D5 is upregulated in LUAD tissues compared to adjacent normal tissues. Furthermore, high expression of SH2D5 is associated with poorer overall survival (OS) and disease-specific survival (DSS) in LUAD patients. Meanwhile, we also found that SH2D5 had abnormal protein expression in LUAD cells compared to normal human bronchial epithelial cells. Then, we investigated the role of SH2D5 in LUAD cells proliferation and metastasis through loss-of-function and gain-of-function assays. We found that overexpression of SH2D5 enhanced the proliferation and metastatic abilities of LUAD cells. In contrast, knockdown of SH2D5 decreased cell viability, migration, and invasion in LUAD cells. The results reveal that SH2D5 may function as an oncogene that is closely associated with the metastasis of LUAD.

Metastasis is the primary cause of cancer-related deaths, as it allows the disease to spread to vital organs and disrupt normal physiological functions. In recent years, numerous studies have demonstrated that the EMT is one of the pivotal processes in driving tumor metastasis [13,29]. Similarly, several studies have shown that the EMT process is involved in the regulation of LUAD cell metastasis [30,31]. Based on the GSEA gene enrichment analysis of the TCGA-LUAD dataset, we found that EMT-related genes were enriched in the SH2D5 high expression group, suggesting that EMT is involved in the regulation of SH2D5 in LUAD cells. Similarly, the western blot analysis shown that overexpression of SH2D5 significantly increased the expression of Snail and Slug, and suppressed E-cadherin; while SH2D5 knockdown correspondingly increased the expression of E-cadherin and repressed the protein levels of Snail and Slug. We noticed that the effect of SH2D5 overexpression on inhibiting E-Cadherin protein expression was not apparent in HCC827 cells, and we speculate that this may be related to its genetic background. Overall, our results suggest that SH2D5 induces a malignant phenotype in LUAD cells, making them more motile and invasive by promoting EMT.

The AKT pathway, also known as the PI3K/AKT/mTOR pathway, is a central hub that integrates diverse extracellular signals and regulates a wide range of cellular processes, including cell proliferation, survival, metabolism, and migration [32,33]. Interestingly, activation of the AKT signaling pathway has been demonstrated to be a key regulatory mechanism controlling EMT in a variety of cancers [18,34,35]. Therefore, we hypothesized that SH2D5 promotes the malignant phenotype of LUAD cells by activating the AKT signaling pathway to regulate the EMT process. As expected, the overexpression of SH2D5 could increase the phosphorylation level of AKT, conversely, SH2D5 knockdown repressed the phosphorylation level of AKT. Following, through rescue experiment, we have demonstrated that SH2D5 exerts its function by regulating the AKT signaling pathway, which in turn leads to the reversal of EMT process. These results demonstrate that SH2D5 regulates EMT through the AKT pathway, thereby affecting the invasion and metastasis of LUAD.

However, our study had some limitations. Our study primarily relied on in vitro experiments, lacking in vivo validation, particularly in the context of metastatic LUAD. This limitation underscores the need for future research to incorporate in vivo models to confirm the relevance of our findings within a physiological environment and to better understand the role of SH2D5 in metastasis. Additionally, our study did not explore the different expression trends of SH2D5 in LUAD cell lines, which is an important aspect for understanding its functional significance in cancer biology. Different LUAD cell lines may exhibit varying expression levels of SH2D5, and these differences could correlate with their metastatic potential or response to treatment. Therefore, further research is needed to explore the internal mechanism of SH2D5 expression trends in LUAD cells.

In conclusion, we found that SH2D5 is aberrantly expressed and associated with poor prognosis in LUAD. Meanwhile, SH2D5 promotes EMT in LUAD cells by activating the AKT signaling pathway, thereby enhancing their metastatic potential. These findings not only enrich the research on the metastatic mechanism of LUAD, but also provide great potential targets for the treatment of metastatic LUAD.

## Supporting information

S1 Raw images (PDF)
