# Supplementary material for: SH2D5 promotes lung adenocarcinoma cell metastasis and triggers EMT via activating AKT signaling pathway
Source: PLoS One. 2024 Dec 30;19(12):e0316432. doi: 10.1371/journal.pone.0316432 (PMC11684657; doi:10.1371/journal.pone.0316432)
Supplement: S1 Raw images — (PDF) [file pone.0316432.s001.pdf]

SH2D5 promotes lung adenocarcinoma cell metastasis and triggers EMT via activating AKT signaling pathway

Western blot raw data

Licheng Du, Wenjia Ren, Linjun Liu, Haojia Zhu, Ke Xu*, Yubai Zhou*

Fig 1E

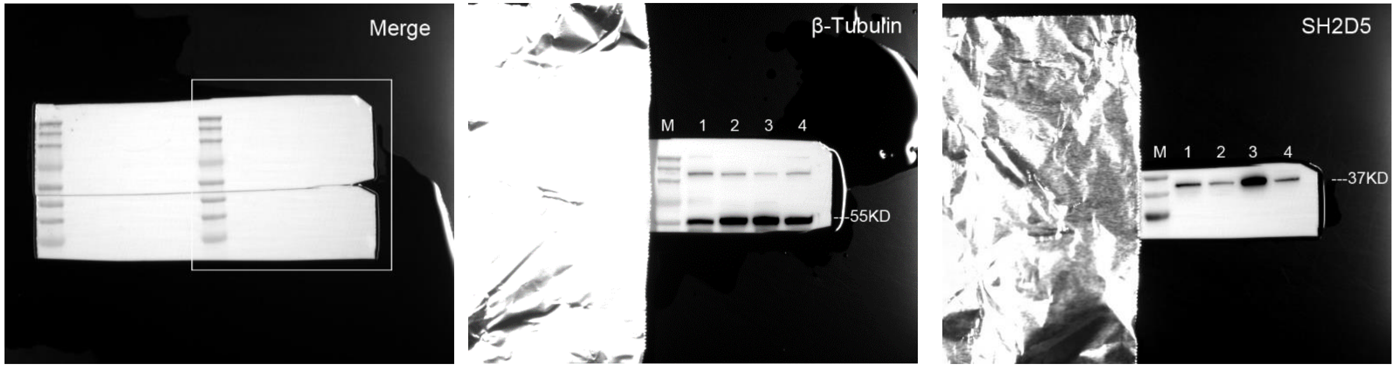

M: Marker (Epizyme, WJ103)

1: 16HBE

2: HCC827

3: H1299

4: A549

Fig 2A HCC827

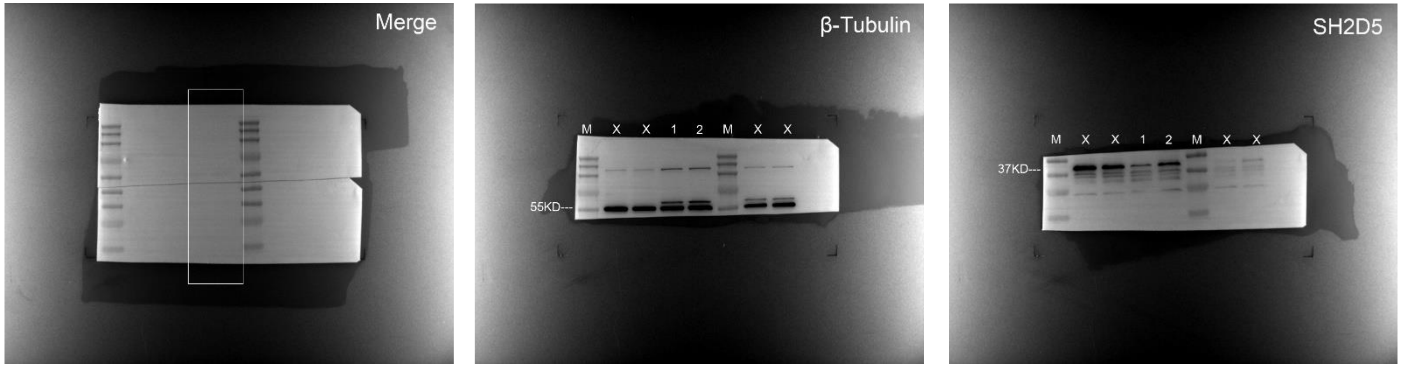

M: Marker (Epizyme, WJ103)

1: HCC827-Vector

2: HCC827-oeSH2D5

Method used to capture the image:

Tanon 5200 Chemiluminescence Imager (Thermo Corporation,USA)

Fig 2A A549

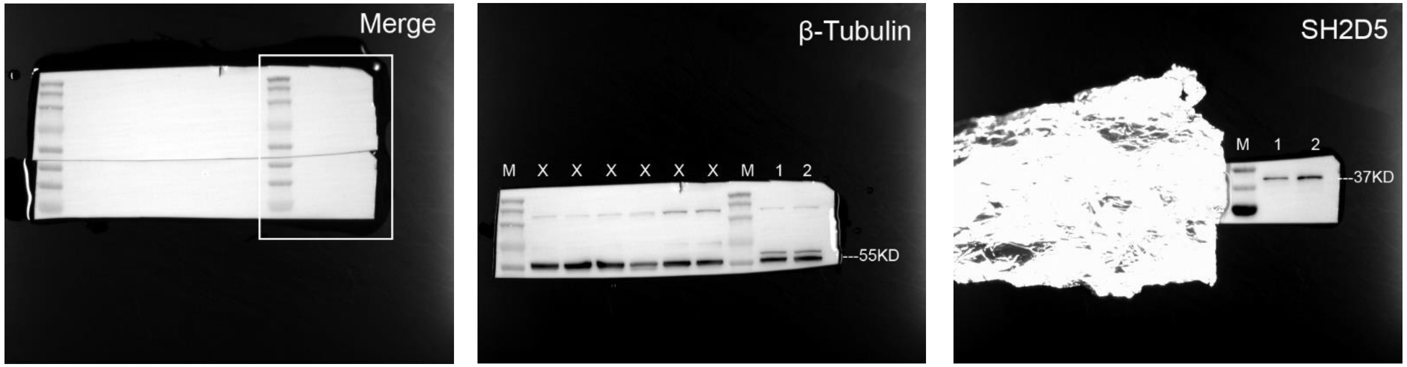

M: Marker (Epizyme, WJ103)

1: A549-Vector

2: A549-oeSH2D5

Fig 3A H1299-Left

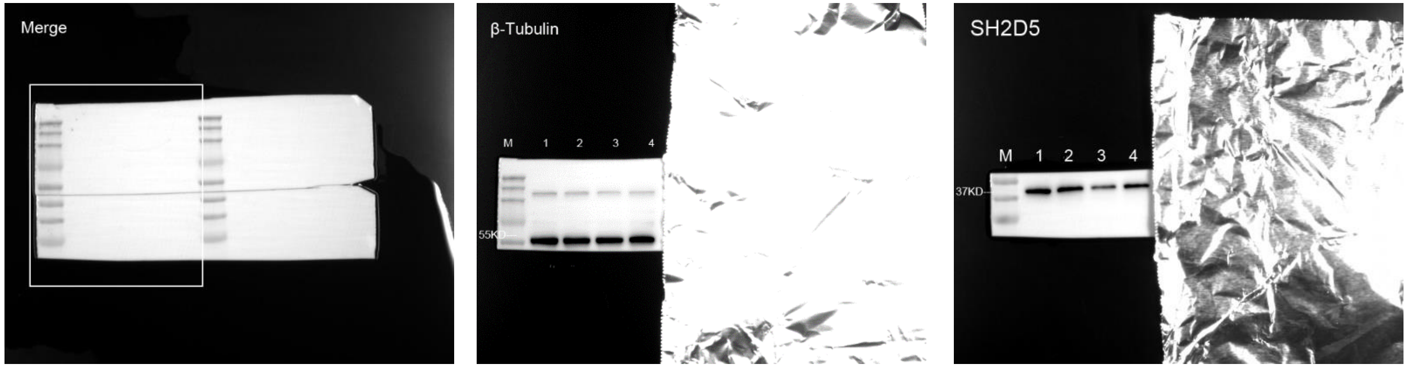

M: Marker (Epizyme, WJ103)

1: H1299-shNC

2: H1299-shSH2D5-1

3: H1299-shSH2D5-2

4: H1299-shSH2D5-3

Method used to capture the image:

Tanon 5200 Chemiluminescence Imager (Thermo Corporation,USA)

Fig 3A H1299-Right

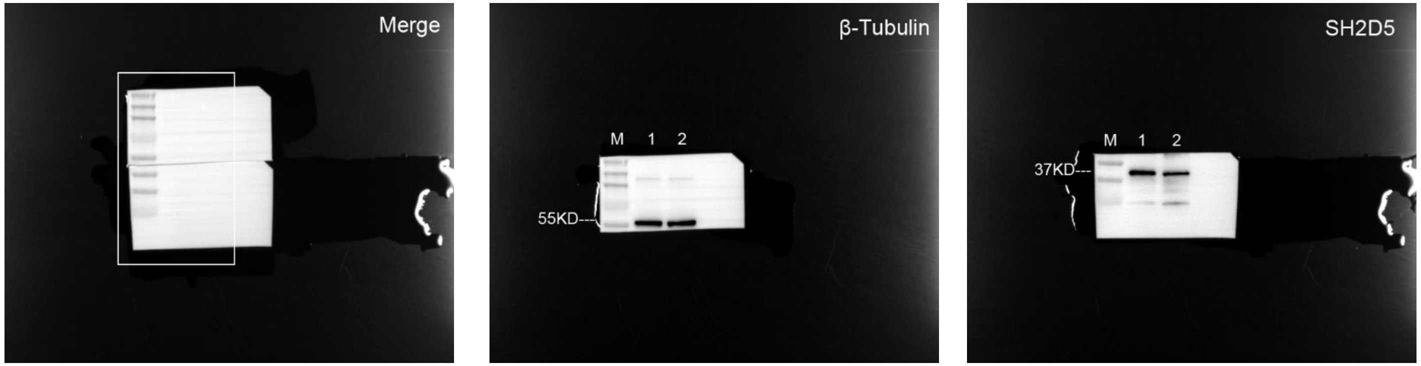

M: Marker (Epizyme, WJ103)

1: H1299-shNC

2: H1299-shSH2D5-2

Fig 4C HCC827

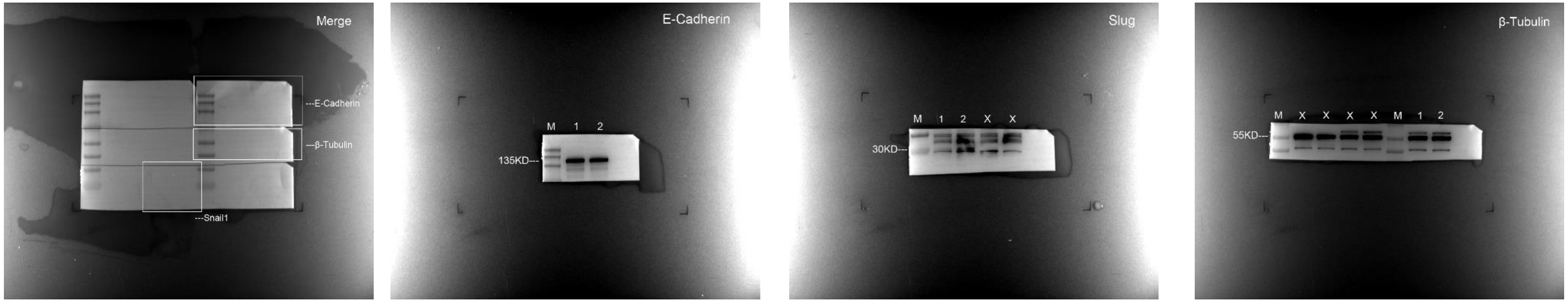

M: Marker (Epizyme, WJ103) / 1: HCC827-Vector / 2: HCC827-oeSH2D5

Method used to capture the image:

Tanon 5200 Chemiluminescence Imager (Thermo Corporation,USA)

Fig 4C HCC827

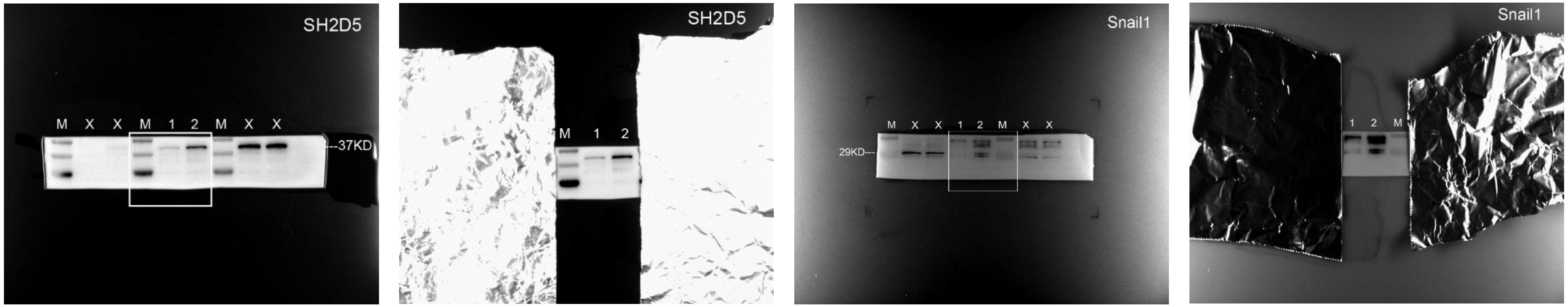

M: Marker (Epizyme, WJ103) / 1: HCC827-Vector / 2: HCC827-oeSH2D5

Fig 4C A549

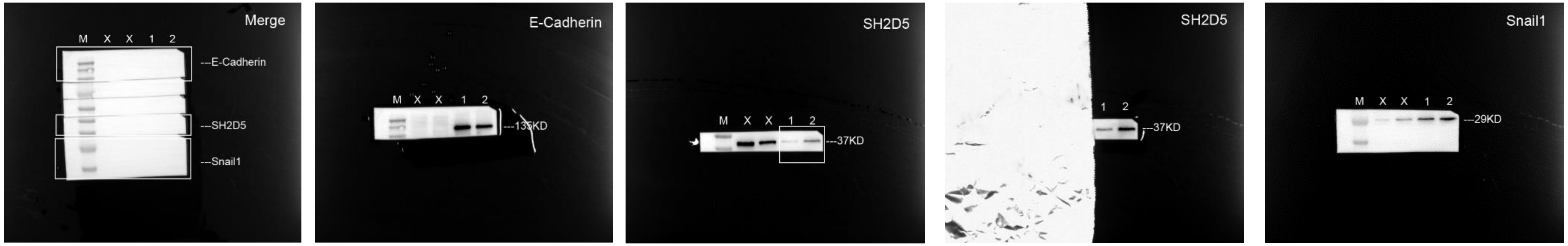

M: Marker (Epizyme, WJ103) / 1: A549-Vector / 2: A549-oeSH2D5

Method used to capture the image:

Tanon 5200 Chemiluminescence Imager (Thermo Corporation,USA)

Fig 4C A549

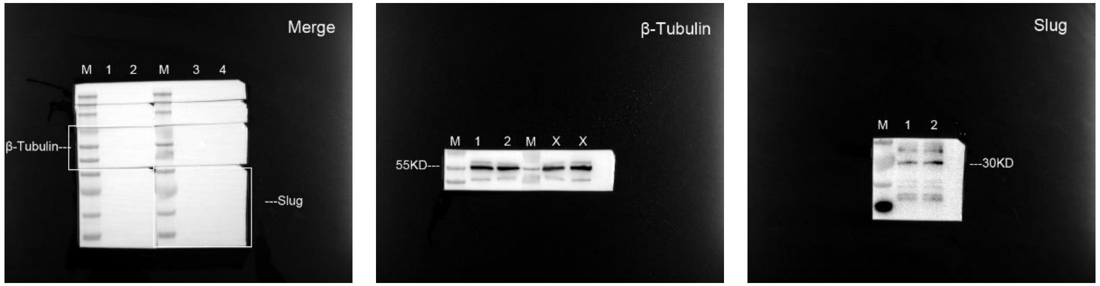

M: Marker (Epizyme, WJ103)
1: A549-Vector
2: A549-oeSH2D5
3: A549-Vector
4: A549-oeSH2D5

Fig 4C H1299

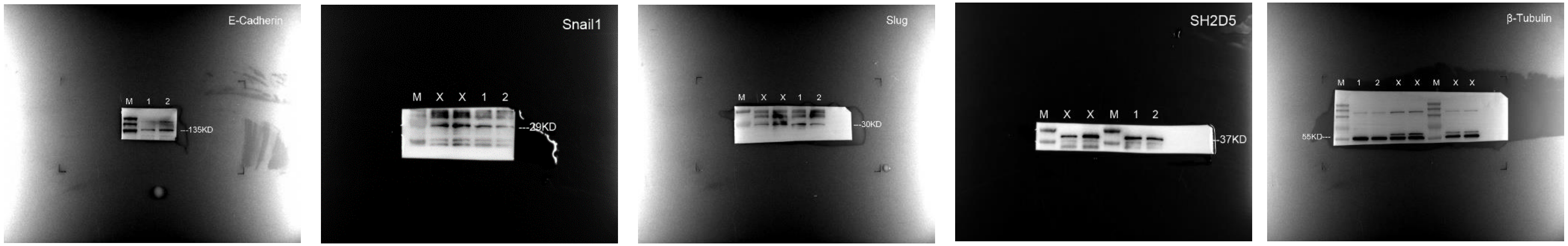

M: Marker (Epizyme, WJ103) / 1: H1299-shNC / 2: H1299-shSH2D5-2

Method used to capture the image:

Tanon 5200 Chemiluminescence Imager (Thermo Corporation,USA)

Fig 5A HCC827

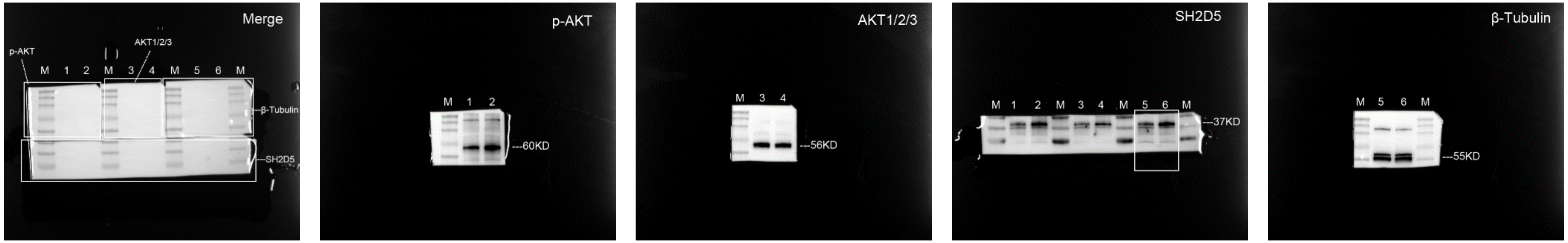

M: Marker (Epizyme, WJ103) / 1: HCC827-Vector / 2: HCC827-oeSH2D5 / 3: HCC827-Vector / 4: HCC827-oeSH2D5 / 5: HCC827-Vector / 6: HCC827-oeSH2D5

Fig 5A A549

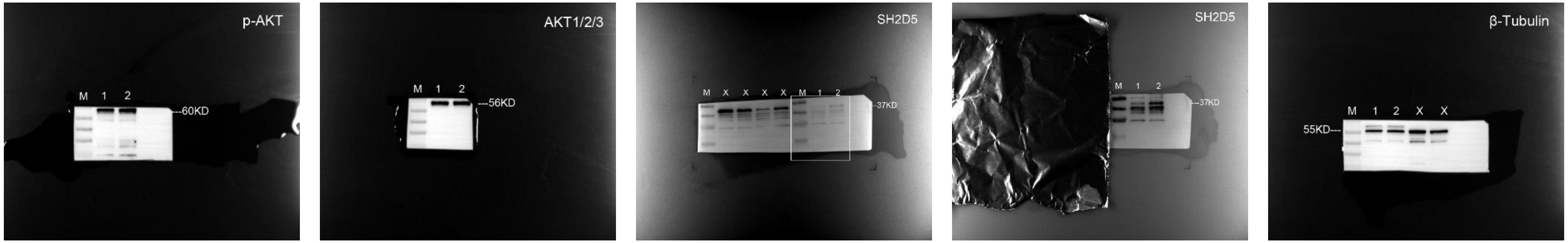

M: Marker (Epizyme, WJ103) / 1: A549-Vector / 2: A549-oeSH2D5

Method used to capture the image:

Tanon 5200 Chemiluminescence Imager (Thermo Corporation,USA)

Fig 5A H1299

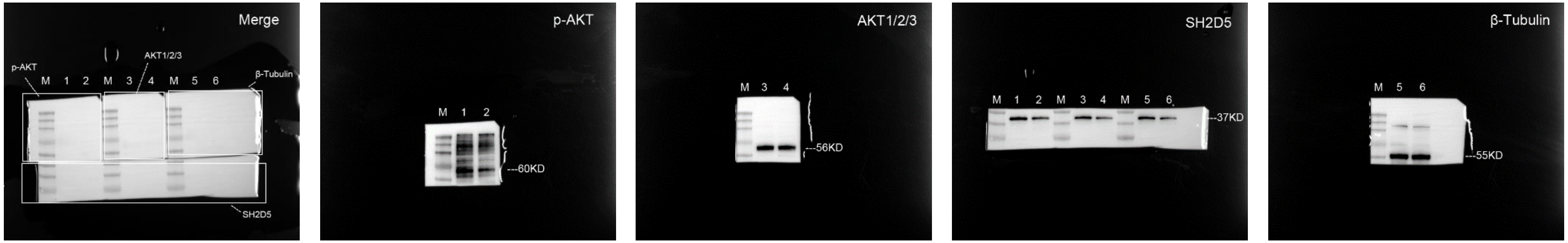

M: Marker (Epizyme, WJ103) / 1: H1299-shNC / 2: H1299-shSH2D5-2 / 3: H1299-shNC / 4: H1299-shSH2D5-2 / 5: H1299-shNC / 6: H1299-shSH2D5-2

Method used to capture the image:

Tanon 5200 Chemiluminescence Imager (Thermo Corporation,USA)

Fig 6G HCC827

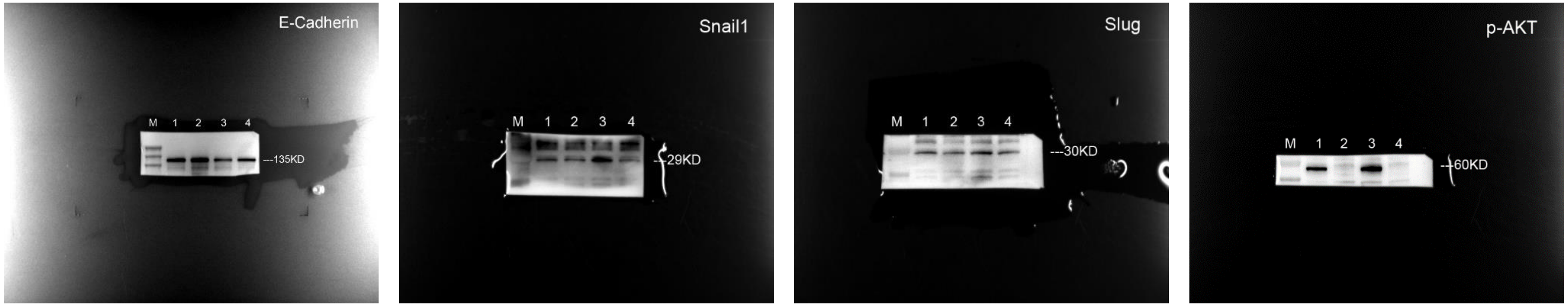

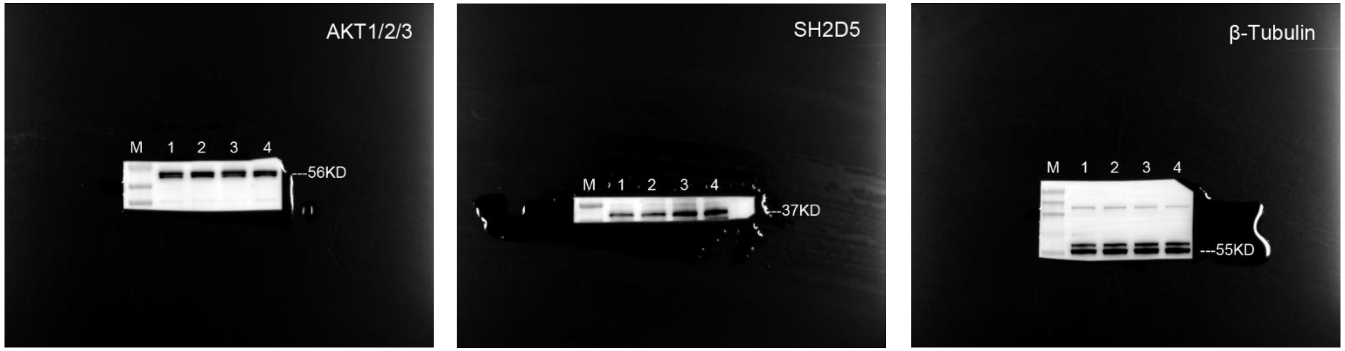

M: Marker (Epizyme, WJ103)

1: HCC827-Vector

2: HCC827-Vector+MK-2206

3: HCC827-oeSH2D5

4: HCC827-oeSH2D5+MK-2206

Method used to capture the image:

Tanon 5200 Chemiluminescence Imager (Thermo Corporation,USA)

Fig 6G A549

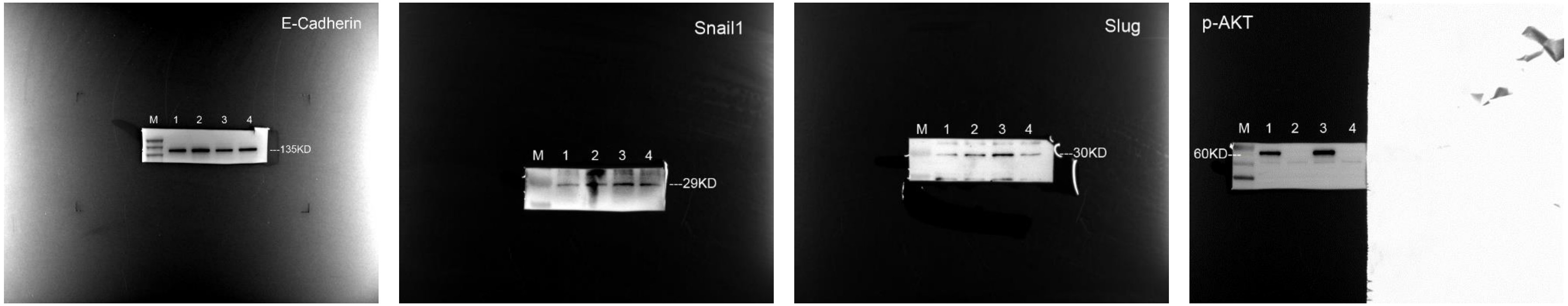

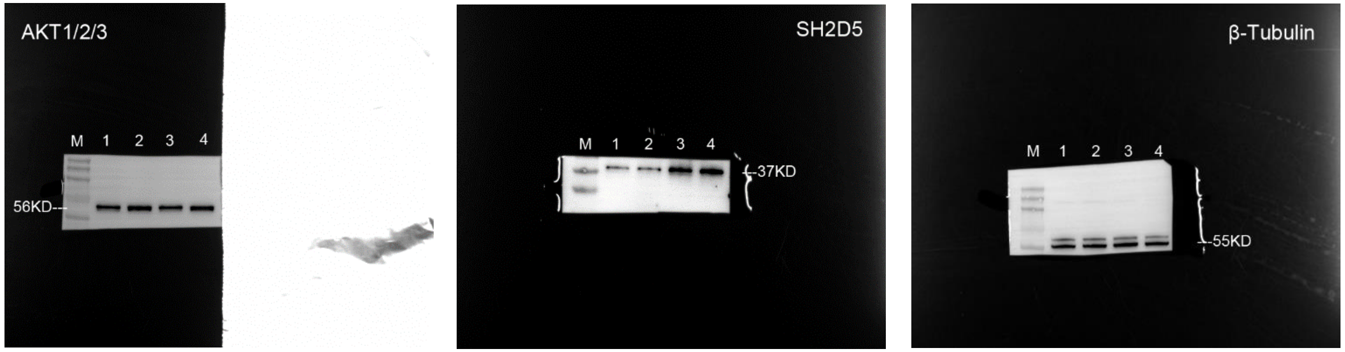

M: Marker (Epizyme, WJ103)

1: A549-Vector

2: A549-Vector+MK-2206

3: A549-oeSH2D5

4: A549-oeSH2D5+MK-2206

Method used to capture the image:

Tanon 5200 Chemiluminescence Imager (Thermo Corporation,USA)

Fig 6G H1299

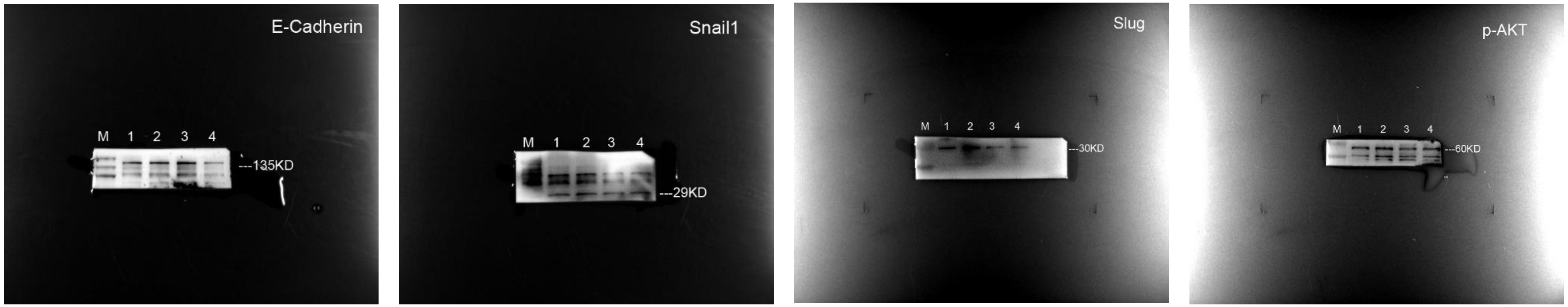

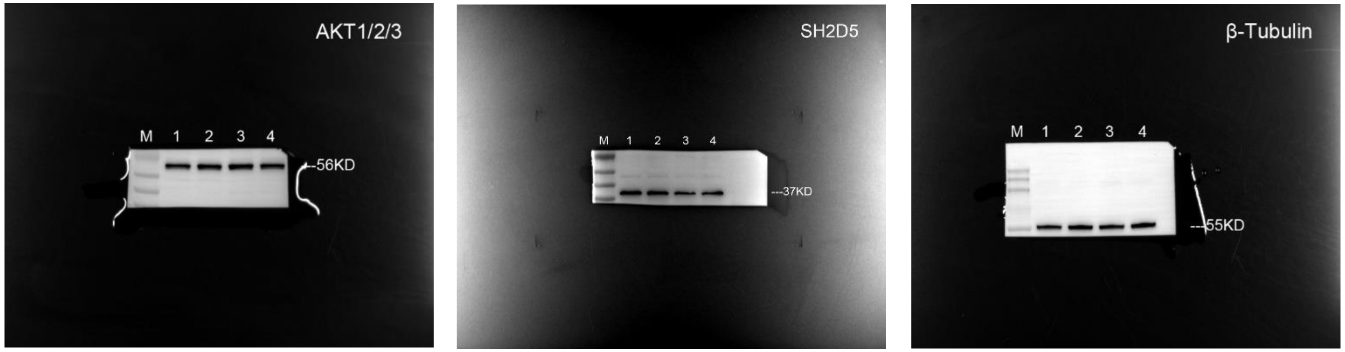

M: Marker (Epizyme, WJ103)

1: H1299-shNC

2: H1299-shNC+SC79

3: H1299-shSH2D5

4: H1299-shSH2D5+SC79

Method used to capture the image:

Tanon 5200 Chemiluminescence Imager (Thermo Corporation,USA)
